# Assessing the Inclusion of Music Therapy and Music Interventions in National Dementia Strategies and Clinical Practice Guidelines: A Scoping Review

**DOI:** 10.3390/healthcare14040511

**Published:** 2026-02-17

**Authors:** Victoria McArthur, Martyn Patel

**Affiliations:** 1 Norfolk and Norwich University Hospital Foundation Trust, Norwich NR7 7UY, UK; 2 Norwich Medical School, University of East Anglia, Norwich NR4 7TJ, UK; martyn.patel@nnuh.nhs.uk

**Keywords:** dementia, acute care, music therapy, clinical practice guidelines, national dementia strategy

## Abstract

Objectives: Dementia prevalence continues to rise, predicted to reach 150 million by 2050, making development of effective, person-centred, non-pharmacological interventions an urgent healthcare priority. Music therapy and music (MTAM) are increasingly recognised as low-cost options to alleviate behavioural and psychological symptoms of dementia (BPSD), particularly in acute hospital environments. This scoping review evaluates national dementia strategies and clinical practice guidelines (CPGs) to determine how far MTAM are acknowledged as formal components of dementia care. Methods: A scoping review of databases identified the most recent national clinical strategies or CPGs for people with dementia (PWD), published between 2015 and 2025. Using the PRISMA guidelines in June 2025, with pre-determined inclusion and exclusion criteria we examined four databases, supplemented with an internet search and reference snowballing. National strategies and CPGs that included MTAM were examined in more detail. Results: Of the 37 national strategies or CPGs, 19 were eligible for inclusion, identified from 16 countries. Although non-pharmacological interventions were widely endorsed, only seven guidelines referenced MTAM, and fewer acknowledged its potential value in hospital. In contrast, interventions such as occupational therapy, reminiscence therapy and cognitive behavioural therapy appeared far more frequently. Further analysis was not in the scope of this review. Conclusions: Our findings underscore the gap between emerging evidence supporting music-based interventions and its inclusion in national policy. Clinical Implications: These findings highlight the need for further robust research demonstrating the benefit of MTAM for PWD to strengthen future policy recommendations and promote integration of music-based approaches in dementia care.

## 1. Introduction

Dementia prevalence is rapidly rising; currently it is estimated that over 55 million people have dementia, and this is predicted to increase to over 130 million cases by 2050 [1]. Dementia is a leading cause of impairment and loss of dependence in adults, affecting cognitive function, mood, ability to perform activities of daily living and independence. Despite advances in research and optimistic future treatments, there is no cure, making strategies to support quality of life, person-centred care and symptom management a global healthcare priority.

The World Health Organization recognizes that dementia is not a single diagnosis or disease but a conceptual term for a syndrome that accompanies a range of neurodegenerative diseases that over time destroys nerve cells, damages the brain, leads to cognitive impairment and affects activities of daily living [2]. Alzheimer’s Disease (AD) is the most common subtype [3], followed by vascular dementia, with Lewy Body dementia (LBD) and Frontal Temporal Dementia (FTD) accounting for a smaller proportion of cases [3,4]. People with dementia (PWD) frequently require hospital care, where unfamiliar environments can exacerbate confusion, anxiety and collectively known as behavioural and psychosocial symptoms of dementia (BPSD) [5]. These behaviours complicate treatment, extend hospital stays and increase healthcare costs [5,6,7,8].

Over the past two decades, there has been a shift away from pharmacological management of BPSD due to limited efficacy and well-documented risks, including increased falls, stroke and mortality [9,10,11,12]. Consequently, clinical guidance now emphasises person-centred, non-pharmacological interventions as first-line approaches [13,14,15,16]. Among these, music therapy and music-based interventions (MTAMs) have gained increasing attention. Music therapy is a recognised clinical intervention delivered by trained professionals, while structured and informal music interventions may also be implemented by caregivers following appropriate training [17,18].

Evidence suggests that MTAM can reduce anxiety and distress, enhance engagement, and support emotional and cognitive functioning in PWD [19,20,21,22]. Although much of the literature focuses on community and residential care settings, there is growing interest in the potential role of MTAM within acute and hospital-based care environments. National dementia strategies and clinical practice guidelines (CPGs) play a critical role in translating such evidence into policy and practice, shaping care delivery and resource allocation.

However, it remains unclear to what extent MTAM are explicitly recognised or recommended within national dementia strategies and dementia CPGs, particularly in relation to acute care. The purpose of this scoping review was therefore to map the inclusion of MTAM within current national dementia strategies and clinical practice guidelines, with a specific focus on their role in supporting person-centred care for people with dementia in acute healthcare settings.

We chose to conduct a scoping review because the available evidence, guidelines and national strategies had not previously been delineated with reference to MTAM. This follows a systematic approach to delineate the extent of the evidence in the literature with the aim of identifying the primary concepts and disparities in knowledge. This review seeks to determine how prominently MTAM features within current national dementia strategies and dementia CPGs, in particular in acute care. This is with a view to raising awareness for those working with PWD in acute healthcare settings of the benefits of using MTAM to alleviate distress and anxiety.

## 2. Materials and Methods

The reporting of this scoping review was guided by the standards of the Preferred Reporting Items for Systematic Review and Meta-Analysis (PRISMA) Statement [23] and followed the PRISMA ScR guidelines [24]. A protocol for this scoping review is available on Open Science Framework, https://osf.io/4upj7/ (accessed on 1 July2025).

This scoping review of national dementia strategies and CPGs was carried out in June 2025. We used a triple search approach. Firstly, four databases were searched (CINHAL, EMBASE, Medline, PsychINFO) with the search terms (see below). To identify further material, an internet search engine, Google, performed targeted searches using specific country names paired with key search terms from the database search list. Thirdly, “reference snowballing” was used; guidelines and frameworks mentioned in other reports, such as those found via Alzheimer’s Europe and other government health ministries, were followed.

The terms used for the database search were as follows:

“Dementia”

“Guideline*”

“Clinical practice guideline”

“Strategy”

“National strategy”

“Action plan”

Inclusion criteria for guidelines were as follows:

- Worldwide national-level guidelines or strategies applicable to dementia care (including guidelines that span multiple neighbouring countries and ones for independently governed Overseas Territories).
- Published or updated since 2015.
- Addressed dementia care comprehensively (not limited to a single symptom, profession or setting).
- Endorsed by a national government, professional body or healthcare authority.
- Legally able to be translated into practice.

Guidelines were excluded under the following criteria:

- Focused only on mild cognitive impairment.
- Exclusive recommendations for one issue (e.g., pain, wandering, diagnosis).
- Directed solely at one healthcare practitioner (e.g., radiographers, medics or nurses).

The search was limited to guidelines published in English (or with an official translation available, no AI technology was used). Both authors screened the identified strategies and guidelines for inclusion. No disagreements occurred, strategies not meeting criteria (symptom profession or setting) were easily identifiable. An assessment of quality was not within the scope of this review due to the nature of the material not being primary research. Results were tabulated on the basis on inclusion of MTAM and acute care, it was also noted if other non-pharmacological therapies were included. Studies meeting the inclusion criteria were examined further by author one, and data extracted and tabulated based on the details of inclusion of MTAM and the key findings of each were checked by the second author.

## 3. Results

**Figure 1:**
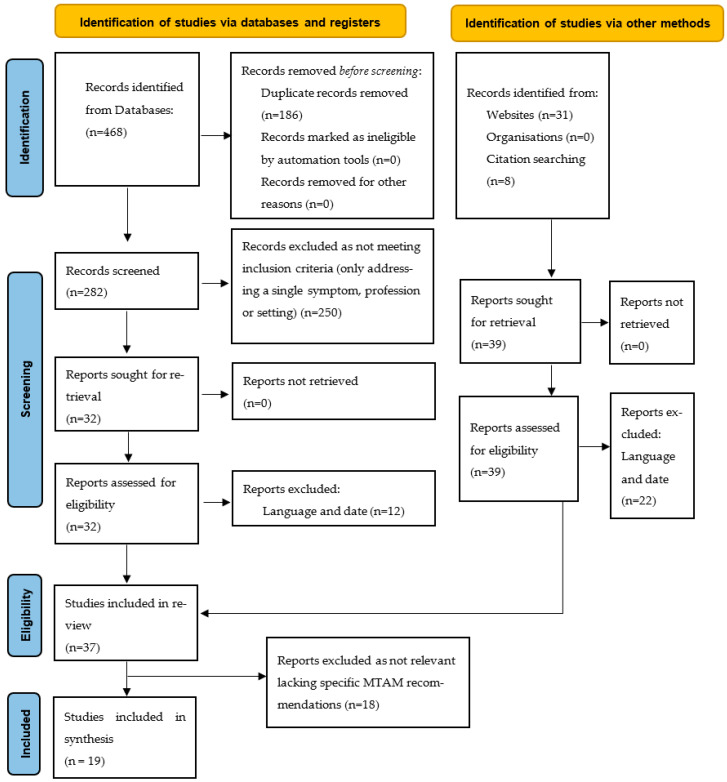
Modified PRISMA diagram [23]. Licensed under CC BY 4.0. (https://creativecommons.org/licenses/by/4.0/, accessed on 16 February 2026).

A total of 37 national dementia strategies and/or dementia clinical practice guidelines from 31 countries were identified. Twelve were excluded because they were not available in English, four excluded because they did not meet the date criteria and two excluded as not deemed relevant. The 19 meeting the inclusion criteria came from 16 countries. The process of study identification is illustrated in a modified PRISMA flow diagram (Figure 1). PRISMA checklist is shown in [25].

As of June 2025, the following countries do not have publicly accessible National Dementia strategies or plans freely accessible: Belgium-Wallonia, Bosnia and Herzegovina, Bulgaria, Cyprus, Estonia, Hungary, Jersey, Latvia, Lithuania, Montenegro, North Macedonia, Poland, Romania, Serbia, Slovakia, Turkey, Ukraine (Alzheimer Europe [26]).

Many guidelines were excluded as they did not describe multifaceted approaches, focusing instead on narrow domains or single issues within dementia care, for example, palliative care or pain management. Some did not relate to dementia care, hence the limited number of accessed and included strategies. The included CPG and strategies are shown in Table 1.

The details of the seven strategies/guidelines from six countries are shown in Table 2.

## 4. Discussion

This review demonstrates that despite the strong shared commitment of national dementia strategies to person-centred, ethical and non-pharmacological care, there is limited recognition of MTAM within these policy frameworks. Of the nineteen meeting the inclusion criteria only seven strategies promoted MTAM for PWD. Despite robust alignment on principles such as dignity, autonomy, empowerment and holistic care for PWD, MTAM remain under-represented in national strategies and CPGs compared with other non-pharmacological approaches.

The findings of this review should be considered in the context of the broader evidence base for MTAM across neurological populations. Music engages preserved neural networks involved in emotion, memory and attention, which may explain its capacity to reduce anxiety, agitation and distress even in advanced neurodegenerative conditions [46]. These mechanisms are particularly relevant to people with dementia, for whom familiar sensory stimuli can support communication, emotional regulation and person-centred care in environments that are otherwise disorientating, such as acute hospital settings.

### 4.1. Areas of Consensus

Within the included documents there was overall consensus on maintaining the dignity and empowerment of PWD, promoting equality and human rights. PWD should be involved in decisions about their care with the best information and advocacy. Emphasis was universally placed on the need for education to reduce stigma and improve public awareness, to inform of risks and possible prevention strategies. Most guidelines promoted dementia-friendly communities where individuals with dementia and their families can participate in a dementia-friendly society, living active and meaningful lives.

Living well with dementia needs person-centred, holistic and coordinated care; this takes work force development and training. Specific support for individuals with dementia and their carers, who are taking most of the burden, should be a routine part of a dementia diagnosis. Many guidelines promoted living at home for as long as possible, reducing burden on residential care. To support carers in this, strategies agreed that community engagement and continuous assessment were critical.

All plans noted the need for training and workforce development. Capacity should be built into health and social care for the future challenges of increasing numbers of PWD. Recognition that dementia is individual and unpredictable, both at a daily level and in its trajectory, requires training in the changing nature of support and care needed. Most guidelines and CPGs supported the need for gathering dementia data and increased research and innovation.

The strong emphasis across national strategies on dignity, autonomy, empowerment and person-centred care aligns closely with the proposed mechanisms of MTAM. By facilitating emotional expression, social connection and meaningful engagement, MTAM practically places these ethical principles in everyday care. The limited explicit reference to MTAM within guidelines represents a missed opportunity to translate widely endorsed values into practical, evidence-informed interventions.

### 4.2. Music Therapy and Music

The focus of this review was to assess the inclusion of MTAM in national guidelines and CPGs as a method of supporting management and person-centred care of PWD. Despite the shared values and consensus, this review found there were significant gaps in how national plans incorporate MTAM. Nearly all of the included guidelines recognised the need for non-pharmacological interventions, and many other therapies were supported as alternative practice in social and health care environments. However, MTAM were rarely acknowledged in detail; seven out of the nineteen included strategies referred briefly to music. Playlist for Life [47] was mentioned in two plans (Belgium and Scotland [30,44]); however, in one case this was more in the context of raising the profile of the individual with dementia rather than as a therapy to aid care.

Further evidence to support the inclusion of MTAM in national strategies comes from other neurological populations. MT has demonstrated beneficial effects across a range of neurological disorders, including improvements in emotional wellbeing, anxiety, sleep, cognitive function and quality of life [48,49]. There are emerging delivery models, such as telecoaching and teletherapy which support adherence, physical functioning and engagement in care, that could be transferable to acute care situations [50]. This cross-condition applicability and feasibility of music-based interventions in complex neurological populations strengthens their case for inclusion in dementia strategies and CPGs, particularly given the shared challenges related the cognitions mental health and functional decline.

Few strategies linked MTAM to acute care, and hospital-based care was covered in only half the included plans. This highlights the need for national strategies to recognise that given the age demographic of PWD, they are more likely to present at acute and emergency care and need support. Research is also clear on the effects of hospitalisation on PWD: increased anxiety, non-compliance and poorer outcomes, with longer hospital stays or mortality often the consequence.

Evidence from both dementia and wider neurological populations suggests that MTAM can support emotional well-being, reduce anxiety and enhance engagement through activation of preserved musical memory and affective processing pathways [21,51,52]. While much of the dementia literature has focused on residential and community settings, emerging evidence from acute care indicates that MTAM may also improve care processes, including reduced resistance to care and enhanced patient–staff interaction [53]. These mechanisms are particularly relevant in hospital environments, where sensory overload and unfamiliar routines commonly exacerbate behavioural and psychological symptoms of dementia.

The distinction between formal music therapy delivered by registered professionals and informal music-based interventions facilitated by trained staff or caregivers has important implications for policy and implementation [17]. A tiered approach, in which professional music therapy is available for individuals with complex needs alongside structured, staff-led music interventions embedded in routine care, may enhance feasibility and equity. However, national strategies and CPGs rarely provide guidance on how such models could be implemented, contributing to variability in practice.

### 4.3. Dementia Subtypes

Whilst there is much research on “AD only” and “all dementia” cohorts, there is relatively less specifically for LBD or FTD. These subtypes present with distinct cognitive, behavioural and sensory features that may influence how individuals respond to interventions such a MTAM. The under-representation of rarer subtypes in MTAM research reflects well-recognised methodological and ethical challenges, including small population sizes and difficulties in recruitment [54,55,56]. This may partly explain the reluctance of policymakers to include MTAM in national guidelines intended to be broadly applicable. Importantly, interventions aimed at reducing distress and supporting emotional well-being may be relevant across dementia subtypes, despite differences in cognitive or behavioural presentation.

This review highlights the importance of recognizing and reporting dementia sub-type when designing and conducting research. Ensuring adequate representation across the dementia subtypes will allow findings to be more inclusive, applicable and evidence-based to support the care of all PWD.

### 4.4. Barriers to the Use of Music Therapy and Music Interventions

Encouragingly, most CPG and national strategies acknowledge the limited benefits of drug therapies for the BPSD and the risks associated with using them and ask for more support for non-pharmacological interventions. The Canadian dementia strategy [31,32] calls for more support for non-drug therapies and names MT as a promising approach, yet there are barriers to implementing MTAM in acute care. Staff shortages, time pressures and limited access to training are all routine occurrences in these environments and more generally over all hospitals. The call for increased training in dementia was universal in all the included CPG and national strategies. As dementia is recognized and stigma reduced, there is a need to increase support, skills training and education across all areas caring for PWD.

Although MTAM are relatively low-cost and low-risk interventions, their implementation in acute care settings is constrained by systemic factors, including time pressures, staffing limitations, and limited access to specialist training. The absence of clear policy endorsement may further impede uptake, particularly in settings dominated by biomedical models of care. Without explicit guidance within national strategies and CPGs, MTAM risk being perceived as optional rather than integral to high-quality dementia care.

Increasingly, hospitals are recognizing the value of multi-professional teams; collaborations within these healthcare teams are leading to improvements in standards of care, particularly with vulnerable patient groups like PWD, yet the prevailing medical dominance, the perceived view that physicians’ viewpoints are prioritized over those of allied health professionals, nurses or other health professionals, can result in slow adoption of non-medical interventions. This applies both in hospitals and in government policies. Strengthening interdisciplinary collaboration and recognising the expertise of allied health professionals are critical to advancing the integration of MTAM into routine practice. As healthcare systems increasingly adopt team-based models of care, formal recognition of music therapy and music-based interventions within policy frameworks could support cultural change and facilitate broader implementation.

MTAM and other therapies will continue to build evidence-based results to support the care of PWD, and as parity of clinical expertise recognized, we have confidence that these therapies will be adopted at administrative and policy level. Although the economic benefits of using MTAM and other therapies to reduce BPSD in acute care makes a strong case for its inclusion in national and local guidance, the most powerful argument is the benefit to caring for PWD.

### 4.5. Limitations and Strengths

This review of the inclusion of MTAM in national dementia guidelines, frameworks and strategies has several limitations which should be considered when interpreting the findings. Guidelines were only selected from those either freely available in English or where a translation existed, and some translations were abbreviated compared to the source document. While this ensured consistency of analysis and transparency, it may have resulted in the exclusion of relevant policy documents published solely in national languages. Consequently, some countries with well-developed dementia frameworks may be underrepresented and the absence of MTAM interventions in certain strategies should not be interpreted as evidence that such approaches are not recognised or used in practice.

National strategies might also be limited in what information they included, and it is possible that sub-national strategies might specifically address the use of music. As a result, this review may underestimate the extent to which non-pharmacological interventions, including MTAM, are embedded in local or service-level practice.

The notable absence of Low- and Middle-Income Countries (LMICs) in this scoping review likely reflects wider structural and systemic barriers in research capacity, health system infrastructure and policy prioritisation in these settings, rather than lack of need. Many LMICs face constrained funding and resources for public health research, making it difficult to generate evidence to drive national dementia strategies and policy development. Nationally representative data on dementia prevalence, costs, and effective interventions can be sparse or methodologically limited. Additionally, competing health priorities can reduce political impetus for formal national plans, particularly when other communicable and non-communicable diseases dominate the policy agenda. These factors contribute to the observed absence of LMIC representation in the dementia strategy literature and to a generally fragmented research base on mental health issues among older adults [57].

The analysis also focused on whether MTAM were mentioned, and it was not within the scope to evaluate the depth, quality or implementation of those recommendations. This approach is consistent with the scoping review objective, mapping the presence or absence of interventions across policy documents, rather than appraising policy quality or effectiveness. Finally, although emergency and acute care were of key interest, many national strategies do not differentiate care settings, and this may limit the specificity of conclusions for hospital-based practice.

Alongside these limitations, this a review has several important strengths. The use of a scoping review methodology enabled a systematic and transparent mapping of the presence of MTAM across a diverse range of national dementia strategies and CPGs. This allowed mapping of heterogeneous evidence to clarify key concepts, identify gaps in knowledge and capture policy level patterns that are not accessible through effectiveness-focused reviews. By examining international frameworks and explicitly considering care settings, acknowledging dementia subtypes and non-pharmacological interventions, this review provides a novel, policy-relevant contribution and establishes a robust foundation for future research, implementation studies and guideline development in this emerging area.

### 4.6. Future Research Directions

Future research should focus on strengthening the evidence base for MTAM across the dementia spectrum, with particular attention to under-represented dementia subtypes and acute hospital settings. Longitudinal and comparative studies are needed to examine clinical, psychosocial and economic outcomes, as well as implementation feasibility within complex care environments. In parallel, policy-focused research exploring how MTAM can be translated into national frameworks, and CPGs will be essential to support equitable, scalable and sustainable integrations into dementia care.

## 5. Conclusions

MTAM is a promising non-pharmacological intervention for managing BPSD, offering safer alternatives to antipsychotic medications, which carry significant risks in older adults. Despite strong evidence of benefit, MTAM is rarely included in national dementia strategies or clinical guidelines and is especially under-represented in acute care settings where distress, agitation and non-compliance are common. MTAM interventions can support person-centred care, reduce anxiety, improve mood, strengthen identity and help maintain dignity—key ethical priorities across all international dementia care guidelines. Implementation barriers persist in acute environments; staff shortages, time limitations and lack of training often prevent use of MTAM, despite its potential to reduce distress and shorten hospital stays.

## Figures and Tables

**Table 1:** Included dementia care national strategies and clinical practice guidelines.

Country	National Strategy	Government/Professional Clinical Practice Guideline	Title and Date of Latest Publication	Music Therapy or Music Intervention Mentioned	Acute or Emergency Care Mentioned	Other Psychological or Social Care Interventions
Australia [27]	YES		National Dementia Action Plan 2024–2025	NO	YES	Physiotherapy, Occupational Therapy (OT), Speech and Language therapy, social workers, dieticians, psychologists, other environmental therapies
Australia [28]		YES	Aged Care Diversity Framework. 2017	NO	NO	OT
Austria [29]	YES		Dementia Strategy Living well with dementia 2015 (Updated report 2025 only in German)	NO	NO	General therapies
Belgium-Flanders [30]	YES		Continuing to build a dementia-friendly Flanders together (2016–2019, update only in French)	YES	NO	NO
Canada [31]	YES		A dementia strategy for Canada, Together We Aspire (2019)	YES	YES	Massage, aromatherapy, pet therapy
Canada [32]		YES	The Canadian Coalition for Seniors’ Mental Health Canadian Clinical Practice Guidelines for Assessing and Managing Behavioural and Psychological Symptoms of Dementia (BPSD) (2025)	YES	YES	Cognitive Behavioural Therapy (CBT), physiotherapy, massage, aromatherapy
Cyprus [33]	YES		National Action Plan for treatment of dementia Cyprus 2012–2017	NO	NO	Physiotherapy, OT
Czech Republic [34]	YES		Czech Republic Nation Alzheimer’s Plan 2020–2030	YES	YES	Art, dance, reminiscence, Garden therapy, CBT
Denmark [35]	YES		A safe and dignified life with dementia. National Action Plan on Dementia 2025 (2017)	NO	NO	NO
Gibraltar [36]	YES		Gibraltar National Dementia Strategy 2018–2021	NO	NO	Speech and language therapy, physiotherapy and OT
Greece [37]	YES		Greek National Plan for Alzheimer 2016	NO	NO	Physiotherapy, OT, Speech and Language therapy
All Nordic countries [38]		YES	Nordic Welfare Centre Dementia prevention in the Nordics 2024	NO	NO	NO
Malta [39]	YES		Reaching new heights. National Dementia Strategy for the Maltese Islands 2024–2031	NO	YES	Complementary therapy
Netherlands [40]	YES		Dutch National Dementia Strategy 2021–2030	NO	NO	NO
Norway [41]	YES		Norway Dementia Plan 2025	YES	YES	Person-centred therapy, milieu therapy, physiotherapy
England [42]	YES		Prime Ministers challenge 2020	NO	YES	Cognitive stimulation therapy
UK [43]		YES	NICE Guideline Dementia: assessment, management and support for people living with dementia and their carers Reviewed 2023	YES	NO	Cognitive stimulation therapy, reminiscence therapy, OT, interpersonal therapy.
Scotland [44]	YES		Dementia in Scotland, Everyone’s Story 2023	YES (Playlist for life)	YES	OT, Validation, Reminiscence therapies
Wales [45]	YES		Dementia Action Plan for Wales 2018–2022	NO	NO	OT, Speech and Language therapy

**Table 2:** Key details of the music interventions mentioned for dementia care in national strategies or clinical practice guidelines.

Strategy/Guideline	Role of Music Therapy or Intervention	Key Details
Continuing to build a dementia-friendly Flanders together (2016–2019) [30]	-Expertisecentrum Dementie Vlaanderen: webinars exploring music, group singing, choirs, playlists to help PWD. -CDs The Voice of Our Memory, intended for PWD, choirs and caregivers to sing together. -A repertoire for joint singing. -Initiatives to connect PWD (at home and in care homes) and facilitate shared musical expression. -Alzheimer Liga Vlaanderen supports dementia-friendly activities, including music/singing.	-There are no programmes delivered by certified music therapists as part of care featuring in the 2016–2019 dementia strategy. -The strategy refers to non-clinical/cultural/community/social approaches to music (singing, choirs, CDs, cultural participation) rather than clinical interventions. -No evidence of explicit targets, funding, or implementation in the strategy related to MTAM specifically.
Canada: “A Dementia Strategy for Canada: Together We Aspire” (2019) and The Canadian Coalition for Seniors’ Mental Health Canadian Clinical Practice Guidelines for Assessing and Managing Behavioural and Psychological Symptoms of Dementia [31,32]	-MTAM included to improve quality of life and wellbeing of PWD. -The strategy acknowledges the limits of drug therapies for BPSD and the risks associated with them. -It calls for more support for non-drug therapies, and MT is named amongst other therapies. -Activities proposed to develop and test “innovative and effective therapeutic approaches,” including MT as a non-drug intervention.	-The strategy wants to support MTAM, key to improving quality of life for PWD and caregivers. -Emphasis on effectiveness, reaching underserved/rural populations carers and embedding guidelines in care practice. -Funded programs such as Dementia Community Investment (DCI) University of Ottawa. And DELIGHT combining music with exercise. Research into community programs to improve wellbeing, physical, cognitive and social outcomes.
Czech Republic Nation Alzheimer’s Plan (2020–2030) [34]	-NAPAN explicitly includes non-pharmacological interventions (including MT)—to be evaluated for their effect on health and quality of life of PWD. -MT is grouped with other therapies (art therapy, reminiscence, dance therapy, garden therapy, cognitive training, rehabilitation). -MT is considered part of psychosocial and therapeutic-activating non-drug interventions.	-The plan does not provide detailed implementation guidelines, specific funding allocations or targets solely for MTAM. -MT included with research and evaluation topics under non-pharmacological interventions. -Limited description of how MT could be integrated into care pathways (community or hospital settings), or training personnel. There is emphasis on research and evidence generation.
Norway Dementia Plan (2025) [41]	-The plan explicitly includes MTAM among non-pharmacological interventions to be researched and evaluated for their impact on health status and quality of life of PWD. -MT is grouped with other expressivity/therapeutic arts interventions (dance, art therapy, reminiscence etc.), indicating recognition of its potential but not as a fully detailed, mandated service.	-Opportunities for PWD and carers to access social, cultural and physical activities adapted to interests, desires and needs included in the plan. -Cultural participation (including music/singing) encouraged to live an active and meaningful life. -The plan also refers to singing and music for therapeutic purposes to reduce BPSD, psychotropic medication usage, improve relationships and wellbeing. -There are specific projects/research studies: e.g., “Homeside” dementia choirs (Dementia Choir Oslo) and music therapy research for caregivers.
NICE Guideline NG97 (UK, 2018) [43]	-A brief referral to MT as one of the non-pharmacological activities that promotes wellbeing. -MT not treated as a primary core intervention (e.g., for cognition). -Stronger recommendations for therapies like group cognitive stimulation, reminiscence and psychosocial support.	-Person-centred care that prioritises non-drug approaches before medications. -MT is considered a valid option for improving mood, engagement and reducing agitation.
Scotland: “Dementia: Everyone’s Story” (2023) [44]	-Highlights Playlist for Life as a leading music-based intervention (a Scottish charity founded in 2013). -The goal of the playlist is that ‘everyone living with dementia’ has a personally meaningful music playlist, that carers and health/social care professionals know how to use.	-Personalised music playlists to enhance wellbeing, communication, reduce stress and improve engagement for PWD. Playlist for Life is explicitly mentioned in the 2023 strategy as a non-pharmacological intervention that may improve cognition, social engagement and quality of life and decrease stress/distress. -There is evidence of integration in healthcare (NHS Fife). -Reported benefits include reduced agitation; improved wellbeing; social interaction; better mood; reminiscence; improved communication and reduced distress.

## Data Availability

No new data were created or analysed in this study.

## Abbreviations

The following abbreviations are used in this manuscript:

PWD	People with dementia
MTAM	Music therapy and music
MT	Music therapy
HCPC	Health and Care Professions Council
BPSD	Behavioural and Psychosocial Symptoms of Dementia
AD	Alzheimer’s Disease
LBD	Lewy Body Dementia
FTD	Frontal Temporal Dementia

## References

[1] World Health Organization (2022). A Blueprint for Dementia Research. https://www.who.int/publications/i/item/9789240058248.

[2] World Health Organization Dementia. https://www.who.int/news-room/fact-sheets/detail/dementia.

[3] Goodman R.A., Lochner K.A., Thambisetty M., Wingo T.S., Posner S.F., Ling S.M. (2017). Prevalence of dementia subtypes in United States Medicare fee-for-service beneficiaries, 2011–2013. Alzheimer’s Dement..

[4] Wu Y.T., Beiser A.S., Breteler M., Fratiglioni L., Helmer C., Hendrie H.C., Honda H., Ikram M.A., Langa K.M., Lobo A. (2017). The changing prevalence and incidence of dementia over time—Current evidence. Nat. Rev. Neurol..

[5] Sinvani L., Strunk A., Ardito S., Gordon S., Liu Y., Schantz E., Arroon A., Ilyas A., Gromova V., Polokowski A. (2023). Reducing Behavioral and Psychological Symptoms of Dementia in Acutely Ill Patients via Patient Engagement Specialists: A Pilot Feasibility Study. Gerontol. Geriatr. Med..

[6] Johnston M., Wakeling A., Graham N., Stokes F. (2011). Cognitive impairment emotional disorder and length of stay of elderly patients in a district general hospital. Br. J. Med. Psychol..

[7] Fogg C., Griffiths P., Meredith P., Bridges J. (2018). Hospital outcomes of older people with cognitive impairment: An integrative review. Int. J. Geriatr. Psychiatry.

[8] Sommerlad A., Perera G., Mueller C., Singh-Manoux A., Lewis G., Stewart R., Livingston G. (2019). Hospitalisation of people with dementia: Evidence from English electronic health records from 2008 to 2016. Eur. J. Epidemiol..

[9] Kales H.C., Gitlin L.N., Lyketsos C.G. (2015). Assessment and management of behavioral and psychological symptoms of dementia. BMJ Clin. Res..

[10] Mok P.L., Carr M.J., Guthrie B., Morales D.R., Sheikh A., Elliott R.A., Camacho E.M., Van Staa T., Avery A.J., Ashcroft D.M. (2024). Multiple adverse outcomes associated with antipsychotic use in people with dementia: Population based matched cohort study. BMJ.

[11] Nørgaard A., Jensen-Dahm C., Wimberley T., Svendsen J.H., Ishtiak-Ahmed K., Laursen T.M., Waldemar G., Gasse C. (2022). Effect of antipsychotics on mortality risk in patients with dementia with and without comorbidities. J. Am. Geriatr. Soc..

[12] O’Neil M., Freeman M., Christensen V., Telerant R., Addleman A., Kansagara D. (2011). A Systematic Evidence Review of Non-Pharmacological Interventions for Behavioral Symptoms of Dementia.

[13] Cooper C., Mukadam N., Katona C., Lyketsos C.G., Ames D., Rabins P., Engedal K., de Mendonça Lima C., Blazer D., Teri L. (2012). Systematic review of the effectiveness of non-pharmacological interventions to improve quality of life of people with dementia. Int. Psychogeriatr..

[14] Douglas S., James I., Ballard C. (2004). Non-pharmacological interventions in dementia. Adv. Psychiatr. Treat..

[15] Gold K. (2014). But does it do any good? Measuring the impact of music therapy on people with advanced dementia: (Innovative practice). Dementia.

[16] NICE (2019). QS184.

[17] British Association of Music Therapy (BAMT). https://www.bamt.org/music-therapy/what-is-a-music-therapist.

[18] Hofbauer L.M., Rodriguez P.F.S. (2025). Comparing two caregiver-delivered music listening interventions for community-dwelling people with dementia: A randomised controlled crossover pilot trial. Dementia.

[19] McArthur V., Everington S., Patel M. (2024). Effectiveness of music-based interventions in acute care settings for people living with dementia to reduce anxiety and enhance the care experience: A systematic review. Arch. Gerontol. Geriatr. Plus.

[20] Van Der Steen J.T., Smaling H.J., van der Wouden J.C., Bruinsma M.S., Scholten R.J., Vink A.C. (2018). Music-based therapeutic interventions for people with dementia. Cochrane Database Syst. Rev..

[21] Edwards H. (2015). Music mirrors: A resource for communication and reminiscence. Nurs. Resid. Care.

[22] Thompson N., Iyemere K., Underwood B.R., Odell-Miller H. (2023). Investigating the impact of music therapy on two in-patient psychiatric wards for people living with dementia: Retrospective observational study. Br. J. Psychol. Open.

[23] Alzheimer’s Disease International (2023). WhatsYourPlan Campaign Report 2021/2022.

[24] PRISMA. https://www.prisma-statement.org.

[25] Tricco A.C., Lillie E., Zarin W., O’Brien K.K., Colquhoun H., Levac D., Moher D., Peters M.D.J., Horsley T., Weeks L. (2018). PRISMA Extension for Scoping Reviews (PRISMAScR): Checklist and Explanation. Ann. Intern. Med..

[26] Alzheimer Europe. https://www.alzheimer-europe.org/resources/publications.

[27] The National Dementia Action Plan. https://www.aihw.gov.au/reports/dementia/dementia-in-aus/contents/national-policy-response-to-dementia#The-National-Dementia-Action-Plan.

[28] Australian Government Department of Health (2017). Aged Diversity Care Framework.

[29] Living Well with Dementia (Austria). https://broschuerenservice.sozialministerium.gv.at/Home/Download?publicationId=65&attachmentName=Dementia_strategy_Living_well_with_dementia_2019_pdfUA_.pdf.

[30] Updated Dementia Plan for Flanders. https://www.alzheimer-europe.org/sites/default/files/2021-10/Flanders%20Dementia%20Plan%202016-2019%20-%20English_0.pdf.

[31] A Dementia Strategy for Canada. https://alzheimer.ca/en/get-involved/change-minds/canadas-national-dementia-strategy.

[32] Canadian Clinical Practice Guidelines for Assessing and Managing the Behavioural and Psychosocial Symptoms of Dementia (BPSD). https://ccsmh.ca/wp-content/uploads/2024/05/DIGITAL_CCSMH_BPSD-Clinical-Guidelines_May2024_ENG.pdf.

[33] National Action Plan for the Treatment of Dementia Cyprus. https://www.alzheimer-europe.org/sites/default/files/2021-10/Cyprus%20National%20Dementia%20Strategy%202012-2017%20-%20English%20Translation.pdf.

[34] Czech Republic National Alzheimer’s Plan. https://www.alzheimer-europe.org/sites/default/files/2021-10/Czech%20Republic%20National%20Alzheimer%20Plan%202020-2030.pdf.

[35] National Action Plan of Dementia 2025 (Demark). https://www.alzheimer-europe.org/sites/default/files/2021-10/Denmark%20National%20Action%20Plan%202025%20-%20English%20Summary.pdf.

[36] Gibraltar National Dementia Strategy 2023–2028. https://www.alzint.org/u/Gibraltar-dementia-strategy-final.pdf.

[37] Greece National Action Plan for Dementia 2016–2020. https://www.alzheimer-europe.org/policy/national-dementia-strategies/greece?language_content_entity=en.

[38] Dementia Prevention in the Nordics. https://nordicwelfare.org/wp-content/uploads/2024/09/Dementia-prevention-in-the-Nordics-1.pdf.

[39] Reaching New Heights. National Dementia Strategy for the Maltese Islands. https://www.alzheimer-europe.org/sites/default/files/2024-03/maltese_dementia_strategy_2024-2031_-_english.pdf.

[40] Dutch National Dementia Strategy 2021–2030. https://www.alzint.org/u/Netherlands-NationalDementiaStrategy2021-2030.pdf.

[41] Dementia Plan 2025 Norway. https://www.alzheimer-europe.org/sites/default/files/2022-11/Norway%20Dementia%20Plan%202025_0.pdf.

[42] Prime Minister’s Challenge on Dementia 2020, U.K. https://assets.publishing.service.gov.uk/media/5a816d6040f0b62305b8ee55/PM_Dementia-main_acc.pdf.

[43] National Institute of Health and Care Excellence (NICE) Dementia. https://www.nice.org.uk/guidance/ng97.

[44] Dementia in Scotland Everyone’s Story Delivery Plan 2024–2026. https://www.gov.scot/binaries/content/documents/govscot/publications/strategy-plan/2024/02/new-dementia-strategy-scotland-initial-2-year-delivery-plan-2024-2026/documents/dementia-scotland-everyones-story-delivery-plan-2024-2026/dementia-scotland-everyones-story-delivery-plan-2024-2026/govscot%3Adocument/dementia-scotland-everyones-story-delivery-plan-2024-2026.pdf.

[45] Dementia Action Plan 2018–2022 (Wales). https://www.gov.wales/dementia-action-plan-2018-2022.

[46] Edwards H., Oppikofer S., Aschwanden D. (2024). The use of audio-biographical cues in dementia care: A four-year evaluation in Swiss hospitals, care, and domestic homes. Front. Dement..

[47] Playlist for Life. https://www.playlistforlife.org.uk.

[48] Lu M.-J., Chen W.-Y., Li D.-J. (2022). Efficacy of music therapy and predictors of sleep disturbance among patients with chronic schizophrenia: A prospective study. Arch. Psychiatr. Nurs..

[49] Moreu-Valls A., Puig-Davi A., Martinez-Horta S., Kulisevsky G., Sampedro F., Perez-Perez J., Horta-Barba A., Olmedo-Saura G., Pagonabarraga J., Kulisevsky J. (2025). A randomized clinical trial to evaluate the efficacy of cognitive rehabilitation and music therapy in mild cognitive impairment in Huntington’s disease. J. Neurol..

[50] Leale I., Vinciguerra C., Di Stefano V., Brighina F., Battaglia G. (2025). Effectiveness of Telecoaching and Music Therapy in Neurological Disorders: A Narrative Review and Proposal for a New Interventional Approach. Healthcare.

[51] Cheong C.Y., Tan J.A.Q., Foong Y.-L., Koh H.M., Chen D.Z.Y., Tan J.J.C., Ng C.J., Yap P. (2016). Creative music therapy in an acute care setting for older patients with delirium and dementia. Dement. Geriatr. Cogn. Disord. Extra.

[52] Daykin N., Parry B., Ball K., Walters D., Henry A., Platten B., Hayden R. (2018). The role of participatory music making in supporting people with dementia in hospital environments. Dementia.

[53] Thompson N., Odell-Miller H., Pointon C., Underwood B.R., Wolverson E., Hsu M.H. (2025). Music therapy Embedded in the Life of Dementia Inpatient Care (MELODIC) to help manage distress: A mixed methods study protocol for co-designing a complex intervention. Nord. J. Music Ther..

[54] Lu L.-C., Lan S.-H., Lan S.-J., Hsieh Y.-P. (2025). Effectiveness of the Music Therapy in Dementia: A Systematic Review and Meta-Analysis of Randomized Controlled Trials. Dement. Geriatr. Cogn. Disord..

[55] Morrin H., Fang T., Servant D., Aarsland D., Rajkumar A.P. (2018). Systematic review of the efficacy of non-pharmacological interventions in people with Lewy body dementia. Int. Psychogeriatr..

[56] Ridder H.M., Wigram T., Ottesen A.M. (2009). A pilot study on the effects of music therapy on frontotemporal dementia—Developing a research protocol. Nord. J. Music Ther..

[57] Saha I., Sundström C., Kandasamy A., Kraepelien M., Dahiya N., Saha A., Jayaram-Lindström N., Chakrabarti A., Benegal V. (2025). Digital interventions for common mental health problems among older adults in low- and middle-income countries: A scoping review. BMJ Global Health.

